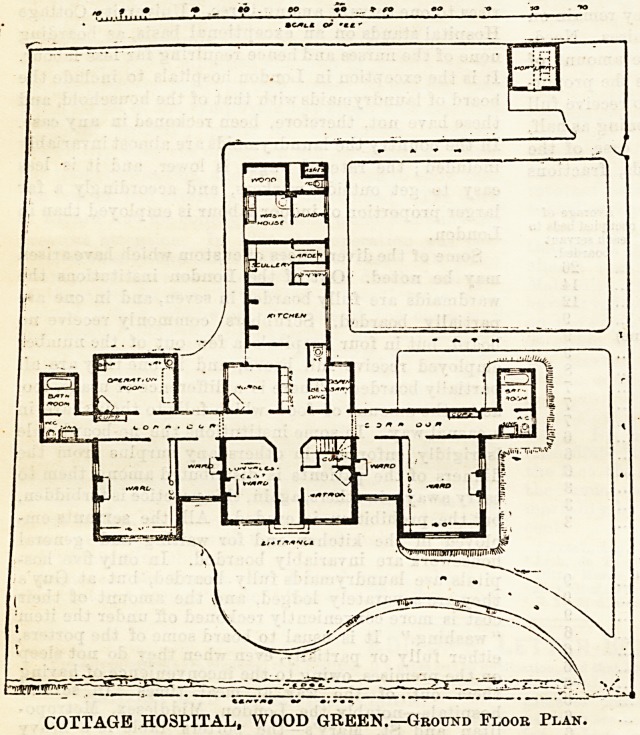# Cottage Hospital, Wood Green

**Published:** 1895-11-09

**Authors:** 


### HOSPITAL CONSTRUCTION.

### COTTAGE HOSPITAL, WOOD GREEN.

This building stands on an open site of one acre, fronting Wood Green Road. The principal entrance is placed between the matron's room and a convalescent ward; passing these rooms corridors branch left and right to the men's and women's departments respectively. One ward, 20 by 16 feet, for four beds, and a small ward for a single bed, are provided for each sex. At the end of each corridor a block containing bath-room, w.c., and sink is cut off by cross-ventilation in the usual way from the main building. Out of the men's corridor opens an operating room, 16 by 11 feet—a not very fortunate position, as it will necessitate all patients from the women's wing being brought for operation past the main entrance and through a portion of the men's corridor.

COTTAGE HOSPITAL, WOOD GREEN.—Ground Floor Plan.

The kitchen wing and nurses' room are well placed in the centre of the building and opposite the entrance. The laundry rooms form a continuation of the kitchen wing, and are entered from the outside. Four bed-rooms are provided for the matron, nurse, and servants over the entrance and rooms adjoining it. The building is undoubtedly a cheap one, but a little more skill might with advantage have been exercised in the preparation of the plans. Thus, no attempt appears to have been made to secure the proper cross lighting and airing of the large wards, or to arrange the doors with a view to the probable position of the beds. Again, to reach the sanitary appliances patients and others must use the main corridor in full view of any person who happens to be in it. The shutting off between two doors of a portion of each corridor, without direct light or air to it, seems a mistake, and the opening of the w.c. (presumably for the matron's and nurses' use) out of a dark and unventilated lobby a serious error.

An extension of the building "on each side for twice the number of patients" is said to be provided for; but in this case the administrative rooms would probably be found quite inadequate in size to meet the wants of the inevitable additions to the working staff.

## Figures and Tables

**Figure f1:**